# Is adult cardiac regeneration absent in *Xenopus laevis* yet present in *Xenopus tropicalis*?

**DOI:** 10.1186/s13578-018-0231-5

**Published:** 2018-04-19

**Authors:** Lindsey Marshall, Fabrice Girardot, Barbara A. Demeneix, Laurent Coen

**Affiliations:** Evolution des Régulations Endocriniennes, Département Adaptation du vivant, UMR CNRS 7221, Muséum National d’Histoire Naturelle, Sorbonne Université, Paris, France

**Keywords:** Anuran amphibians, *Xenopus laevis*, *Xenopus tropicalis*, Cardiac regeneration, Heart injury

## Abstract

We recently used an endoscopy-based resection method to explore the consequences of cardiac injury in adult *Xenopus laevis*, obtaining the result that the adult *Xenopus* heart is unable to regenerate. At 11 months post-amputation, cellular and biological marks of scarring persisted. We thus concluded that, contrary to urodeles and teleosts, adult anurans share a cardiac injury outcome similar to adult mammals. However, in their work published in this journal on the 13 December 2017, Liao et al. showed that the adult *Xenopus tropicalis* heart is capable of efficient, almost scar free regeneration, a result at odds with our previous observation. These findings contrast with and challenge the outcome of adult heart repair following injury in *Xenopus* species. Here we discuss the question of the intrinsic cardiac regenerative properties of an adult heart in anuran amphibians.

Open discussion on the reproducibility of adult *Xenopus* heart regeneration data following apical resection:

Dear Editor,

A key question in cardiovascular biology is to what degree the heart is able to regenerate after tissue damage, and why this capacity varies between evolutionary-separate vertebrate species. If teleost fishes (with the exception of medaka) display high regenerative capacities throughout their entire life, why is mammalian heart regeneration hindered soon after birth, causing adults to lose the ability to regenerate their injured myocardium. In amphibians, evolutionarily positioned between these two phyla, urodeles (i.e. newt, axolotl or salamander) possess lifelong cardiac regenerative capacity like zebrafish, whereas anuran amphibians (i.e. *Xenopus*) remained poorly investigated till recently [1].

In the past year, we and others published contradictory results on the cardiac outcome in adult anuran amphibians after cardiac resection [2, 3]. Following an apical amputation of 4% of the ventricle volume in adult *Xenopus laevis*, we observed significant fibrous scarring, cardiomyocyte hypertrophy and sarcomere disorganisation near the injury site, persisting 11 months post-amputation. Together, these observations allowed us to conclude that the adult heart was unable to regenerate in this species [2]. Conversely, in the closely related species *Xenopus tropicalis,* a cardiac apex resection removing approximately 10% of the adult heart length, showed almost scar-free regeneration in 30–60 days, which provides, according to the authors, “a powerful tool for recapitulating a perfect regeneration phenomenon” [3]. These contrasting results could be interpreted as indicating that cardiac regenerative capacity is differentially distributed in the *Xenopus* genus.

It is not the first time that heart regeneration observations noted in the literature are controversial. Such an example was reported when analysing cardiac regeneration in the MRL/MpJ mouse strain, where some groups observed cardiac regeneration in adult MRL mice whilst others did not, but this discrepancy remains for the moment unresolved (see in [1]).

However, the most pertinent example that has been the cause of confusion and conflicting positions comes from observations in neonate mice, a topic that was “hotly” debated after experimental results showed a different outcome in cardiac rebuilding [4]. In 2011, Porrello et al. [5] published the elegant demonstration that neonatal mice have the potential to fully regenerate resected myocardium in 21 days, whereas a study by Andersen et al. [6] called into question these results. This paper reported limited evidence of regeneration in apically resected neonate hearts at 21 days post-surgery [6]. The conflicting results were partly resolved by Bryant et al. [7], who advanced technical considerations as an important variable that could influence the experimental design, and may explain failure to reproduce the observations between researchers applying a similar cardiac injury protocol. However, the question still remains unanswered, notably in the light of recent work by Zebrowski et al. [8] that fuels the debate. These authors showed that binucleation and cell-cycle arrest occur very soon after birth and are boosted following heart resection in newborn mice [8]. They suggest that if there is a regenerative period in the mammalian neonate, it is very short.

It is well known that mammalian cardiomyocytes become binucleated and polyploid soon after birth, resulting in cell-cycle arrest and hypertrophy in adults [8–10]. In contrast, in zebrafish, cardiomyocytes remain mostly mononucleated even at adult stage, potentially explaining why they continue to be highly proliferative throughout life [10]. While cardiomyocyte nucleation has been poorly investigated during *Xenopus* development, a major difference between *X. laevis* and *X. tropicalis* is that the first has a pseudotetraploid genome and the latter a diploid genome. This difference might be implicated in the opposite outcomes of their cardiac regenerative capacity observed in adults. Potentially, the tetraploid nature of cardiomyocytes in *X. laevis* may render them more prone to polyploidy and hypertrophy. In contrast, *X. tropicalis* could retain a higher concentration of mononucleated cardiomyocytes, similar to zebrafish, which may explain the proliferative response observed in adult heart.

Bryant and co-authors have shown that the resection size influences the extent of scarring and thus the regenerative outcome of the mouse heart [7]. However, we believe that resection size cannot explain the discrepancies between results in *X. tropicalis* observed by Liao and collaborators and our own observations in adult *X. laevis.* They report that their protocol results in the removal of approximately 10% of ventricle length while our approach led to the removal of 4% of the ventricle volume, hence, one would rather expect our protocol to be more favourable to regeneration.

A major difference in our respective approach is the technical methodology applied to perform the resection. We developed a minimally invasive endoscopy-based resection method to explore the consequences of cardiac injury in adult frogs, while in Liao’s work, the cardiac apex was resected using Vannas scissors after externalising the heart. Again, it could be expected that our approach leads to less trauma to cardiac tissue and should also favour regeneration. Nonetheless, we have also performed resection applying a protocol similar to Liao’s to *X. laevis* frogs and observed no evidence of cardiac regeneration (unpublished experiments). Another difference between both techniques was that, following tissue removal using biopsy forceps via an endoscope, we do not apply pressure to the wound in the form of sterile cotton, due to the internal progress of the endoscopic technique. Thus, in our technique, the blood flow immediately following amputation remained internally trapped within the animal. Yet again, we do not think that this modification underlies the different results, as we observed a rapid formation of a large blood clot at the injury site after the biopsy amputation, similar to Liao’s description which mentioned the formation of the clot in few seconds. Furthermore, the clot is limited to the site of amputation as the pericardium is only slightly open at the apex and therefore remains close around the heart during and after the amputation, protecting the heart from the external environment (which is not the case in Liao’s method). Despite these reservations, it is still possible that the methodology, endoscopy versus external heart resection could be implicated in the differential responses.

We consider the critical point could be the methods used to determine the presence (or absence) of cardiac regenerative capacity, notably, how the conclusion of regeneration is obtained (monitoring fibrosis extent, cardiomyocyte hypertrophy, cardiomyocyte cell-cycle activity…) as underlined by Bryant et al. [7], in their discussion on the mouse model. Thus, in our respective work on *Xenopus,* the distinct criteria used to assess the regenerative process, may explain at least in part the observed differences. In our paper, we not only performed histological analyses, but we also made extensive use of immuno-labelling to assess the degree of fibrosis, hypertrophy and the sarcomere structure. All criteria considered converged to the same conclusion that regeneration is totally absent in *Xenopus* hearts in adult frogs even after about 1 year post amputation [2]. While Liao’s work mainly relies on histological staining, it is noteworthy that the fibrosis is indeed absent in most of their samples 60 days post injury [3].

Another disputable point is the methods used by Liao and collaborators to assess cardiomyocyte mitoses, that according to some authors is suggestive of a regenerative capacity [5, 11]. Liao et al. first used hematoxylin/eosin staining. In our opinion, and given the available stringent and more adapted methodology to detect newly formed cardiomyocytes (for instance, using pulse/chase experiments with BrdU incorporation to detect DNA synthesis in cardiomyocyte nuclei [5, 11]), we consider that hematoxylin/eosin staining is not sufficiently precise to follow proliferation during regeneration and risks over estimating the proliferative status of cardiomyocytes. When analysing cardiac regeneration in zebrafish, hematoxylin/eosin staining has been used to observe the tissue as a whole and to show the presence of nucleated blood cells in the damaged area following resection [12]. Thus it cannot be excluded that the proliferative cells identified by Liao et al. using hematoxylin/eosin staining could have represented proliferating non-cardiomyocyte cells, such as erythrocytes or immune cells rather than cardiomyocytes.

Liao et al. also used another method to detect cardiomyocyte mitoses. They performed co-labelling using the cytoplasmic alpha-skeletal muscle actin (α-SMA) antibody to label cardiomyocytes and the pH3 antibody to follow cell cycle activity (proliferation). We are surprised that authors did not use the more appropriate marker Mef2c (Myocyte Enhancer Factor 2C), that specifically labels the cardiomyocyte nuclei, to identify unambiguously the co-localised pH3 positive cardiomyocytes in their samples. Thus, the antibody combination used by Liao et al. to detect proliferative cardiomyocytes can lead to misinterpretation, especially because a number of other non-cardiomyocyte cells, such as blood cells, fibroblasts and epicardial cells also proliferate after cardiac injury.

Transgenic approaches can be also used to specifically label cardiomyocyte nuclei, for instance, using a nuclear localised fluorescent reporter under control of a cardiac specific promoter. Such protocols have been applied to zebrafish, using the cardiac myosin light chain 2 (cmlc2) promoter to differentiate actual cardiomyocytes from non-cardiomyocyte cells, at the amputated site after cardiac resection [13]. A similar approach could be used in *Xenopus*, for instance, by creating transgenic animals with a nuclear fluorescent reporter driven by the cardiac specific promoter pMLC1v [14]. Using such a transgenic approach would allow easy and accurate labelling of cardiomyocyte nuclei. It would thus facilitate the unambiguous identification of the cells that proliferate after cardiac amputation in an adult frog. Furthermore, transgenic animals have also been used to assess the origin of proliferating cardiomyocytes in zebrafish and neonatal mice [5, 11]. Here, an inducible Cre recombinase system driven by a cardiac specific promoter was used to de-repress a reporter construct (GFP in zebrafish; lacZ in mice) blocked by a loxP-flanked transcriptional stop codon, allowing expression of the reporter specifically in resident cardiomyocytes. After amputation, the reporter expression was observed in newly formed cardiomyocytes that replaced the lost myocardial tissue. These demonstrations showed that for neonatal mice and zebrafish, myocardial regeneration is principally achieved by newly formed cardiomyocytes derived from preexisting cardiomyocytes [5, 11]. Applying a similar technology in *Xenopus* seems possible as it has been shown that both inducible system and Cre-based approaches can be used in this species [15, 16]. Developing such methodologies would allow lineage tracing in the heart and allow one to determine the origin of newly formed cardiomyocytes after cardiac amputation in *Xenopus*.

Finally, differences in cardiac regenerative capacity have previously been observed in organisms belonging to the same group. Indeed, in teleosts, zebrafish possess lifelong and highly efficient cardiac regenerative capacity [12] while this competence is not observed in medaka [17]. Therefore, it is not necessarily surprising that closely related *Xenopus laevis* and *tropicalis* may have different and opposed cardiac outcomes following heart resection. In teleosts, distinct activation of the immune response is involved in these differences in heart repair [18]. Remarkably, proper activation of the immune system is also required for cardiac regeneration in the adult urodele amphibian axoltl [19] as well as for determining competence for tail-regeneration and scar-free wound healing in *X. laevis* [20, 21]. It would hence be extremely interesting to explore whether adult *Xenopus laevis* and *tropicalis* species also display differences in their immune responses following cardiac injury.
